# Trends in psychological distress among US adults before, during, and after the COVID-19 pandemic: a repeated cross-sectional analysis, 2017–2024

**DOI:** 10.1016/j.pmedr.2026.103554

**Published:** 2026-06-30

**Authors:** Todd Burus, Tia N. Weber

**Affiliations:** a Markey Cancer Center, University of Kentucky, Lexington, KY, United States; b Division of Biomedical Informatics, College of Medicine, University of Kentucky, Lexington, KY, United States; c Department of Psychiatry, College of Medicine, University of Kentucky, Lexington, KY, United States

**Keywords:** Mental health, Anxiety, Depression, Patient health Questionnaire-4, COVID-19 pandemic

## Abstract

**Objective:** Psychological distress among United States (US) adults following the COVID-19 pandemic remains incompletely characterized. To address this gap, we compared the prevalence of psychological distress during and after the pandemic with pre-pandemic levels using nationally representative data.

**Methods:** We analyzed six iterations of the Health Information National Trends Survey. Psychological distress was measured using the Patient Health Questionnaire-4. Adjusted prevalence ratios (aPRs) comparing early, late, and post-pandemic periods with the pre-pandemic period were estimated using weighted logistic regression models accounting for key demographic and geographic characteristics. The data were collected January 2018 to September 2024 in the US.

**Results:** Among 25,459 respondents, the prevalence of clinically meaningful psychological distress was 12.8% during the pre-pandemic period, 14.7% during the early pandemic period, 14.0% during the late pandemic period, and 14.9% during the post-pandemic period. Compared with pre-pandemic levels, distress prevalence was similar during the early pandemic (aPR = 1.06; 95%CI = 0.83,1.36) and late pandemic (aPR = 1.09; 95%CI = 0.94,1.27) but higher post-pandemic (aPR = 1.16; 95%CI = 1.02,1.32). Increases were most evident for anxiety-related symptoms and among males, younger adults, and college graduates.

**Conclusions:** Psychological distress among US adults was higher in 2024 than before the pandemic, underscoring the need for sustained mental health surveillance, prevention strategies, and resource allocation.

## Highlights

• Psychological distress remained elevated after the COVID-19 pandemic.
• Anxiety most notable among post-pandemic psychological distress symptoms.
• Younger adults and males experienced the largest increases.
• Findings suggest sustained prevention and mental health services are needed.

## 1 Introduction

Psychological distress among US adults increased during the COVID-19 pandemic, but population-level trends remain incompletely characterized, as much of the existing literature has relied on single time points, data limited to the early pandemic period, or inconsistent measures. Early pandemic-related analyses generally observed increases in anxiety and depressive symptoms during the initial months of 2020, with some evidence of attenuation as the pandemic progressed (Ettman et al., 2020; Shuster et al., 2021). Subsequent studies from later pandemic time points reported heterogeneous patterns across population subgroups, including differential changes by socioeconomic status and age (Qian et al., 2023; Jiang et al., 2025; Kim et al., 2023; Inoue et al., 2025). While a study by Arnett and Mitra (Arnett and Mitra, 2025) tracked population-level trends into 2024 and concluded that population-level anxiety and depression remained well above pre-pandemic levels, the use of changing data sources and question presentations raise methodological concerns about the comparability of their findings across different time periods. As a result, it remains unclear whether psychological distress returned to pre-pandemic levels or remained elevated following the end of the COVID-19 public health emergency (*Statement on the fifteenth meeting of the IHR (2005) Emergency committee on the COVID-19 pandemic*, 2023). Using nationally representative survey data spanning from 2017 to 2024, and a consistent and clinically-validated measure of anxiety and depressive symptoms, we assessed whether—and to what extent—the prevalence of psychological distress among US adults differed during and after the COVID-19 pandemic compared with pre-pandemic levels, both overall and within demographic and geographic subgroups.

## 2 Methods

This study was deemed exempt from review and participant consent was waived by the University of Kentucky Institutional Review Board due to the use of de-identified data. We followed the Strengthening the Reporting of Observational Studies in Epidemiology (STROBE) reporting guidelines.

### 2.1 Study Design and Population

We obtained information on anxiety and depression from the National Cancer Institute's (NCI) Health Information National Trends Survey (HINTS) (National Cancer Institute, 2025). The HINTS is a nationally-representative self-report survey of noninstitutionalized, civilian adults developed to monitor changes in health communication. Along with individual behaviors and beliefs related to health information, the HINTS also captures additional information on participants' current mental health status. This study includes data from six iterations of the HINTS survey: HINTS 5 Cycle 1 (2017), HINTS 5 Cycle 2 (2018), HINTS 5 Cycle 3 (2019), HINTS 5 Cycle 4 (2020), HINTS 6 (2022), and HINTS 7 (2024). Surveys had response rates of 32.4%, 32.9%, 30.6%, 37.0%, 28.1%, and 27.3%, respectively. HINTS 5 Cycles 1–4 were offered as mail-in paper surveys only, while HINTS 6 and HINTS 7 were offered on both paper and online.

### 2.2 Measures

Survey periods were classified as being before the COVID-19 pandemic (HINTS 5 Cycles 1–4), during the early COVID-19 pandemic (HINTS 5 Cycle 4), during the late pandemic (HINTS 6), and after the COVID-19 pandemic (HINTS 7). HINTS 5 Cycle 4 was collected from February through June 2020 and included a variable indicating whether individual responses were completed before or after the WHO identified COVID-19 as a global pandemic on March 11, 2020 (*Statement on the fifteenth meeting of the IHR (2005) Emergency committee on the COVID-19 pandemic*, 2023).

We measured psychological distress among respondents according to the Patient Health Questionnaire-4 (PHQ-4) (Kroenke et al., 2009). The PHQ-4 is a valid and reliable self-reported instrument used to screen for anxiety and depression with four questions: “Over the past 2 weeks, how often have you been bothered by: (1) Little interest or pleasure in doing things?; (2) Feeling down, depressed or hopeless?; (3) Feeling nervous, anxious or on edge?; and (4) Not being able to stop or control worrying?” Valid responses include “not at all”, “several days”, “more than half the days”, and “nearly every day”, scored from 0 to 3. A total PHQ-4 score of ≥6 represents clinically meaningful psychological distress. PHQ-4 responses can be further broken into subscales for anxiety alone (questions 3 and 4) and depression alone (questions 1 and 2), with subscale totals of ≥3 representing a positive screening for anxiety or depression, respectively. We created binary variables for clinically meaningful psychological distress from the full PHQ-4 and positive indications of anxiety or depressive disorders alone from the two subscales.

Previous literature has demonstrated the validity and reliability of the PHQ-4 at evaluating clinically meaningful psychological distress among the general population, making it an ideal tool for assessing population-level mental health in a nationally-representative survey (Löwe et al., 2010). While an earlier population-level study (Kessler et al., 2022) found a slight increase in anxiety and depression among US adults during the first year of the COVID-19 pandemic, these results were based on a single question assessed to have a strong correlation with the PHQ-4 and not the PHQ-4 instrument itself. The use of the complete questionnaire in the HINTS administration gives us confidence that our findings adhere to the established psychometric properties for screening of psychological distress among respondents.

We also collected information on respondent sex (male or female), age, race and ethnicity, educational attainment, urbanicity, and Census region residence (Northeast, Midwest, South, and West). Age was stratified into five groups: aged 18–34 years, 35–49 years, 50–64 years, 65–74 years, and ≥ 75 years. Race and ethnicity were self-reported and classified as Hispanic, non-Hispanic Asian, non-Hispanic Black, non-Hispanic White, and other non-Hispanic races. Other non-Hispanic races included American Indian or Alaska Native, Native Hawaiian or other Pacific Islander, and multiple races, and were combined due to low counts. Educational attainment was classified as no college education, some college education, or college graduate. Urbanicity was defined according to the Rural-Urban Continuum Codes (RUCC) as metropolitan (RUCC 1–3) or nonmetropolitan (RUCC 4–9) (U.S. Department of Agriculture, Economic Research Service, 2020).

### 2.3 Statistical analysis

We merged the six HINTS datasets together to explore trends in psychological distress across the COVID-19 pandemic with appropriate estimates for uncertainty. Datasets were merged according to the replicate variance estimation method using the *survey* package (version 4.4–2) in the R statistical programming language following guidance provided by the NCI (National Cancer Institute, 2025). Only respondents with complete data on the dependent variables and each covariate were included in the final analysis. A sensitivity analysis was performed on primary outcomes using hot deck imputation of missing values (Andridge and Little, 2011).

We estimated the prevalence of clinically meaningful psychological distress and positive indications of anxiety or depressive disorders across time periods. Adjusted prevalence ratios (aPR) with 95% confidence intervals (CI) were estimated using logistic regression with average marginal predictions, with adjustments made for sex, age, race and ethnicity, educational attainment, urbanicity, and Census region (Graubard and Korn, 1999). Educational attainment was used as a proxy for household income due to high levels of missing income data (11.6% of sample). Mode of survey administration was considered for adjustment but removed due to collinearity with the period variable. Multicollinearity among other covariates was ruled out using variance inflation factor. Additional prevalence ratios were estimated for each included covariate.

Differences were assessed at *P* < 0.05, and all hypotheses were two-sided. Subgroup analyses were exploratory and were not adjusted for multiple comparisons; as a result, all subgroup-specific findings should be interpreted as hypothesis-generating. All analyses were performed in the R statistical programming language (version 4.4.1; R Foundation).

## 3 Results

The HINTS contained responses from 29,622 US adults across 6 iterations between January 2017 and September 2024. We removed 4163 records having missing data for at least one of the dependent variables or covariates, resulting in a final analytic dataset of 25,459 respondents (Supplementary Fig. 1). Among these, 11,733 (weighted 55.0%; 95% CI, 54.4%,55.6%) were from the pre-pandemic period, 2076 (10.9%; 95% CI, 10.4%,11.5%) were from the early pandemic period, 5418 (16.9%; 95% CI, 16.6%,17.2%) were from the late pandemic period, and 6232 (17.1%; 95% CI, 16.9%,17.4%) were from the post-pandemic period. Additional characteristics of the final analytic dataset are available in Table 1.

**Table 1:** Sample characteristics by period, Health Information National Trends Survey, 2017–2024. Pre-pandemic period includes HINTS 5 Cycle 1 (2017), HINTS 5 Cycle 2 (2018), HINTS 5 Cycle 3 (2019), and HINTS 5 Cycle 4 (February 1–March 11, 2020). Early Pandemic period includes HINTS 5 Cycle 4 (March 12–June 30, 2020). Late Pandemic period includes HINTS 6 (2022). Post-pandemic period includes HINTS 7 (2024). Abbreviations: Prop = weighted proportion.

	Pre-pandemic		Early Pandemic		Late Pandemic		Post-pandemic	
	N	Prop (95% CI)	N	Prop (95% CI)	N	Prop (95% CI)	N	Prop (95% CI)
Overall	11,733	55.0 (54.4,55.6)	2076	10.9 (10.4,11.5)	5418	16.9 (16.6,17.2)	6232	17.1 (16.9,17.4)
Sex								
Female	6827	50.4 (49.8,50.9)	1207	51.8 (49.7,53.9)	3259	50.9 (50.0,51.8)	2491	51.3 (50.5,52.0)
Male	4906	49.6 (49.1,50.2)	869	48.2 (46.1,50.3)	2159	49.1 (48.2,50.0)	3741	48.7 (48.0,49.5)
Age Group								
Aged 18–34 years	1539	24.0 (22.5,25.5)	321	31.1 (28.2,34.0)	811	25.2 (23.4,27.1)	1042	25.6 (23.7,27.5)
Aged 35–49 years	2349	27.1 (25.6,28.7)	471	28.3 (25.1,31.6)	1106	25.8 (23.9,27.8)	1314	26.8 (25.0,28.6)
Aged 50–64 years	3831	30.7 (29.6,31.8)	632	24.3 (22.1,26.6)	1585	28.1 (26.7,29.5)	1632	26.5 (25.2,27.9)
Aged 65–74 years	2538	11.1 (10.8,11.4)	445	10.3 (9.3,11.4)	1212	13.0 (12.6,13.4)	1344	12.7 (12.3,13.1)
Aged ≥75 years	1476	7.1 (6.8,7.4)	207	6.1 (5.4,6.9)	704	7.9 (7.5,8.3)	900	8.4 (8.1,8.8)
Race and Ethnicity								
Hispanic	1700	15.7 (15.2,16.2)	398	18.8 (16.9,20.9)	962	16.8 (16.3,17.2)	1248	17.0 (16.5,17.5)
Non-Hispanic Asian	528	5.2 (4.9,5.6)	107	5.8 (4.5,7.2)	284	5.8 (5.4,6.3)	334	5.6 (5.1,6.2)
Non-Hispanic Black	1552	10.3 (9.9,10.8)	316	12.5 (11.1,14.1)	858	11.0 (10.6,11.4)	937	11.1 (10.7,11.5)
Non-Hispanic White	7522	65.5 (64.9,66.1)	1187	60.2 (58.1,62.2)	3134	61.8 (60.8,62.8)	3475	61.1 (60.4,61.8)
Other Non-Hispanic Races	431	3.2 (2.9,3.5)	68	2.7 (1.9,3.7)	180	4.6 (3.6,5.8)	238	5.2 (4.9,5.5)
Education								
No College	2695	28.6 (27.7,29.6)	518	30.0 (27.2,33.0)	1270	27.6 (26.1,29.2)	1375	26.8 (25.3,28.2)
Some College	3504	38.4 (37.4,39.5)	603	40.0 (36.8,43.2)	1550	39.2 (37.8,40.6)	1807	38.5 (37.0,40.1)
College Graduate	5534	33.0 (32.5,33.5)	955	30.0 (28.1,31.8)	2598	33.2 (32.6,33.8)	3050	34.7 (34.1,35.3)
Urbanicity								
Metropolitan	10,294	86.5 (85.5,87.5)	1879	88.8 (86.1,91.2)	4725	87.7 (86.8,88.7)	5374	87.0 (85.9,88.1)
Nonmetropolitan	1439	13.5 (12.5,14.5)	197	11.2 (8.8,13.9)	693	12.3 (11.3,13.2)	858	13.0 (11.9,14.1)
Census Region								
Northeast	1775	17.5 (17.0,18.1)	293	16.7 (14.9,18.5)	799	17.6 (17.0,18.2)	894	17.5 (17.0,17.9)
Midwest	2096	20.9 (20.4,21.5)	337	20.0 (18.0,22.2)	922	21.4 (20.9,21.9)	1065	21.3 (20.9,21.8)
South	5009	37.6 (36.9,38.2)	928	38.9 (36.4,41.5)	2449	38.3 (37.5,39.1)	2839	38.2 (37.5,39.0)
West	2853	24.0 (23.5,24.6)	518	24.4 (22.3,26.6)	1248	22.6 (21.7,23.6)	1434	22.9 (22.2,23.6)

### 3.1 Prevalence of psychological distress

**Fig. 1:**
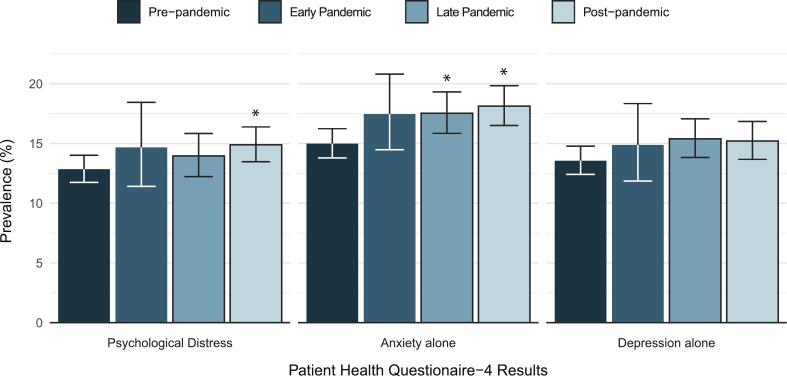
Prevalence of clinically meaningful psychological distress among US adults by period, Health Information National Trends Survey, 2017–2024. Prevalence of clinically meaningful psychological distress (Patient Health Questionnaire-4 score ≥ 6), positive indicators for anxiety disorders (anxiety subscale score ≥ 3), and positive indicators for depressive disorders (depression subscale score ≥ 3). An asterisk (*) represents a period in which the adjusted prevalence ratio for that period compared to the pre-pandemic period is significantly greater than 1, representing a significant increase. Model adjustments were made for sex, age group, race and ethnicity, educational attainment, urbanicity, and Census region. The pre-pandemic period included 11,733 responses, the early pandemic included 2076, the late pandemic included 5418, and the post-pandemic period included 6232.

During the pre-pandemic period, respondents had a mean PHQ-4 score of 2.1 (standard error, 0.05), with an estimated 12.8% having clinically meaningful psychological distress (95% CI, 11.7%,14.0%) (Fig. 1 and Supplementary Table 1). The mean PHQ-4 score during the early pandemic period rose to 2.3 (0.13) with a psychological distress prevalence of 14.7% (95% CI, 11.4%,18.4%). Late pandemic PHQ-4 scores also had a mean of 2.3 (0.06) with a psychological distress prevalence of 14.0% (95% CI, 12.2%,15.8%). Post-pandemic respondents had a mean PHQ-4 score of 2.4 (0.08) and a 14.9% prevalence of psychological distress (95% CI, 13.5%,16.4%). The prevalence of positive indications of anxiety disorders according to the anxiety subscale were 15.0% (95% CI, 13.8%,16.2%), 17.5% (95% CI, 14.5%,20.8%), 17.5% (95% CI, 15.8%,19.3%), and 18.1% (95% CI, 16.5%,19.8%) across the four periods, respectively. Likewise, the prevalence of positive indications of depressive disorders according to the depression subscale were 13.6% (95% CI, 12.4%,14.8%), 14.9% (95% CI, 11.9%,18.3%), 15.4% (95% CI, 13.8%,17.1%), and 15.2% (95% CI, 13.7%,16.8%).

Prior to the COVID-19 pandemic, the prevalence of clinically meaningful psychological distress was lowest among individuals aged 65–74 years (7.2%; 95% CI, 5.9%,8.7%), individuals aged ≥75 years (8.1%; 95% CI, 6.1%,10.5%), and college graduates (8.3%; 95% CI, 6.9%,9.9%), and highest among individuals aged 18–34 years (17.2%; 95% CI, 13.9%,21.0%) and individuals of other non-Hispanic race and ethnicity (17.4%; 95% CI, 10.0%,27.4%). Psychological distress was more prevalent among females than males during the pre-pandemic period (females, 15.4% [95% CI, 14.0%,17.0%]; males, 10.2% [95% CI, 8.6%,12.0%]; *P* < 0.001), similar during the early pandemic period (females, 16.3% [95% CI, 12.0%,21.4%]; males, 12.9% [95% CI, 8.7%,18.2%]; *P* = 0.27) and late pandemic period (females, 15.2% [95% CI, 13.1%,17.5%]; males, 12.7% [95% CI, 10.1%,15.6%]; *P* = 0.13), and more prevalent among males than females during the post-pandemic period (females, 12.6% [95% CI, 9.8%,15.9%]; males, 17.3% [95% CI, 15.5%,19.2%]; *P* = 0.03) (Supplementary Table 1). The prevalence of psychological distress generally decreased with increasing age and increasing educational attainment across all periods.

### 3.2 Comparison of psychological distress between periods

Adjusting for population differences, the prevalence of clinically meaningful psychological distress was unchanged between the pre-pandemic period and early (aPR, 1.06; 95% CI, 0.83,1.36) and late (aPR, 1.09; 95% CI, 0.94,1.27) pandemic periods (Table 2), but 16% higher during the post-pandemic period (1.16; 95% CI, 1.02,1.32). Considered separately, indications of anxiety disorders were similar between the pre-pandemic and early pandemic periods (aPR, 1.08; 95% CI, 0.89,1.32), 18% more prevalent during the late pandemic period (1.18; 95% CI, 1.04,1.33) and 21% more prevalent during the post-pandemic period (1.21; 95% CI, 1.07,1.36). The prevalence of individuals with a positive screening for depressive disorders was similar across all four periods. Results from sensitivity analysis with hot deck imputation were slightly attenuated, though significantly increased prevalence of psychological distress and positive indications of anxiety disorders remained during the post-pandemic period (Supplementary Table 2).

**Table 2:** Comparison of Patient Health Questionnaire-4 (PHQ-4) results to pre-pandemic period among US adults, Health Information National Trends Survey, 2017–2024. Adjusted prevalence ratio (aPR) of PHQ-4 results during the COVID-19 pandemic and post-pandemic periods compared to the pre-pandemic period, adjusted for sex, age, race and ethnicity, educational attainment, urbanicity, and Census region residence. Pre-pandemic period includes HINTS 5 Cycle 1 (2017), HINTS 5 Cycle 2 (2018), HINTS 5 Cycle 3 (2019), and HINTS 5 Cycle 4 (February 1–March 11, 2020). Early Pandemic period includes HINTS 5 Cycle 4 (March 12–June 30, 2020). Late Pandemic period includes HINTS 6 (2022). Post-pandemic period includes HINTS 7 (2024). Abbreviations: CI = confidence interval; PHQ-4 = Patient Health Questionnaire-4.

	Early Pandemic (aPR, 95% CI)	Late Pandemic (aPR, 95% CI)	Post-pandemic (aPR, 95% CI)
**Status**			
Clinically meaningful psychological distress (PHQ-4 ≥ 6)	1.06 (0.83,1.36)	1.09 (0.94,1.27)	1.16 (1.02,1.32)
Positive anxiety disorder (anxiety subscale ≥3)	1.08 (0.89,1.32)	1.18 (1.04,1.33)	1.21 (1.07,1.36)
Positive depressive disorder (depression subscale ≥3)	1.03 (0.83,1.29)	1.14 (0.99,1.30)	1.13 (0.98,1.29)

### 3.3 Comparison of psychological distress among subgroups between periods

Among population subgroups examined, only college graduates reported a higher prevalence of psychological distress during the early and late COVID-19 pandemic periods than in the pre-pandemic period (early pandemic aPR, 1.55 [95% CI, 1.20,2.20]; late pandemic aPR, 1.43 [95% CI, 1.14,1.80]) (Table 3). In contrast, a higher prevalence of psychological distress was reported during the post-pandemic period among males (1.69; 95% CI, 1.39,2.06), individuals aged 18–34 years (1.40; 95% CI, 1.08,1.80), individuals of non-Hispanic White race and ethnicity (1.22; 95% CI, 1.01,1.48), individuals with no college education (1.32; 95% CI, 1.05,1.65), and college graduates (1.36; 95% CI, 1.08,1.71). Roughly 1-in-4 individuals aged 18–34 years reported clinically meaningful psychological distress during the post-pandemic period (24.7%; 95% CI, 20.9%,28.7%).

**Table 3:** Changes in psychological distress by US adult subgroups, Health Information National Trends Survey, 2017–2024. Adjusted prevalence ratio (aPR) of individuals with Patient Health Questionnaire-4 score ≥ 6 during the COVID-19 pandemic and post-pandemic periods compared to the pre-pandemic period, adjusted for sex, age, race and ethnicity, educational attainment, urbanicity, and Census region residence. Pre-pandemic period includes HINTS 5 Cycle 1 (2017), HINTS 5 Cycle 2 (2018), HINTS 5 Cycle 3 (2019), and HINTS 5 Cycle 4 (February 1–March 11, 2020). Early Pandemic period includes HINTS 5 Cycle 4 (March 12–June 30, 2020). Late Pandemic period includes HINTS 6 (2022). Post-pandemic period includes HINTS 7 (2024). Abbreviations: CI = confidence interval.

Group	Early Pandemic (aPR, 95% CI)	Late Pandemic (aPR, 95% CI)	Post-pandemic (aPR, 95% CI)
Sex			
Female	0.99 (0.73,1.35)	0.99 (0.84,1.17)	0.82 (0.64,1.07)
Male	1.16 (0.78,1.72)	1.24 (0.94,1.63)	1.69 (1.39,2.06)
Age Group			
Aged 18–34 years	1.06 (0.65,1.74)	1.27 (0.94,1.72)	1.40 (1.08,1.80)
Aged 35–49 years	1.20 (0.82,1.75)	1.22 (0.91,1.64)	1.15 (0.83,1.59)
Aged 50–64 years	0.94 (0.63,1.38)	0.85 (0.67,1.08)	1.04 (0.81,1.33)
Aged 65–74 years	1.03 (0.59,1.79)	0.89 (0.61,1.30)	0.84 (0.56,1.25)
Aged ≥75 years	1.23 (0.63,2.41)	0.84 (0.52,1.35)	1.09 (0.66,1.78)
Race and Ethnicity			
Hispanic	0.85 (0.54,1.36)	1.10 (0.77,1.55)	1.19 (0.92,1.54)
Non-Hispanic Asian	0.66 (0.21,2.08)	0.80 (0.30,2.11)	0.65 (0.26,1.62)
Non-Hispanic Black	0.90 (0.51,1.57)	1.17 (0.80,1.71)	1.08 (0.77,1.53)
Non-Hispanic White	1.19 (0.89,1.59)	1.11 (0.89,1.37)	1.22 (1.01,1.48)
Other Non-Hispanic Races	1.71 (0.71,4.12)	0.95 (0.45,1.99)	1.01 (0.51,1.99)
Education			
No College	1.07 (0.69,1.65)	1.02 (0.78,1.33)	1.32 (1.05,1.65)
Some College	0.87 (0.60,1.27)	1.00 (0.77,1.29)	0.98 (0.80,1.20)
College Graduate	1.55 (1.10,2.20)	1.43 (1.14,1.80)	1.36 (1.08,1.71)
Urbanicity			
Metropolitan	1.09 (0.84,1.42)	1.05 (0.89,1.24)	1.14 (0.98,1.33)
Nonmetropolitan	0.79 (0.44,1.42)	1.29 (0.95,1.76)	1.29 (0.89,1.86)
Census Region			
Northeast	1.00 (0.53,1.91)	1.25 (0.85,1.85)	1.08 (0.77,1.52)
Midwest	1.24 (0.74,2.09)	1.02 (0.74,1.39)	1.14 (0.81,1.60)
South	1.02 (0.72,1.45)	1.10 (0.88,1.38)	1.22 (0.98,1.51)
West	1.02 (0.66,1.59)	1.02 (0.74,1.41)	1.07 (0.82,1.40)

Anxiety disorder indicators were more prevalent during the early pandemic period than the pre-pandemic for individuals aged 35–49 years (1.39; 95% CI, 1.01,1.93) and college graduates (1.35; 95% CI, 1.02,1.79) (Supplementary Table 3). During the late pandemic period, positive indications of anxiety disorders were more prevalent among males (1.27; 95% CI, 1.02,1.58), individuals aged 35–49 years (1.38; 95% CI, 1.08,1.76), college graduates (1.47; 95% CI, 1.22,1.76), and metropolitan residents (1.17; 95% CI, 1.02,1.34). Compared to pre-pandemic levels, post-pandemic indications of anxiety disorders were more prevalent among males (1.77; 95% CI, 1.48,2.12), individuals aged 18–34 years (1.39; 95% CI, 1.10,1.75), individuals of Hispanic ethnicity (1.32; 95% CI, 1.04,1.68), individuals of non-Hispanic White race and ethnicity (1.23; 95% CI, 1.03,1.46), individuals with no college education (1.37; 95% CI, 1.12,1.69), college graduates (1.42; 95% CI, 1.15,1.75), metropolitan residents (1.20; 95% CI, 1.05,1.37), and residents of the South Census region (1.21; 95% CI, 1.00,1.47).

Positive indications of depressive disorders increased during the pandemic period among college graduates (1.44; 95% CI, 1.01,2.05); during the late pandemic period among individuals aged 18–34 years (1.53; 95% CI, 1.14,2.05), college graduates (1.47; 95% CI, 1.21,1.79), and residents of the Northeast Census region (1.42; 95% CI, 1.02,1.99); and during the post-pandemic period among males (1.35; 95% CI, 1.08,1.67), individuals aged 18–34 years (1.39; 95% CI, 1.05,1.86), and college graduates (1.33; 95% CI, 1.06,1.67) (Supplementary Table 4).

## 4 Discussion

To our knowledge, this study provides one of the first nationally representative assessments of psychological distress among US adults using a consistent, clinically-validated measurement across the pre-COVID-19 pandemic, early and late pandemic, and post-pandemic periods. In this repeated cross-sectional analysis, we found that the prevalence of clinically meaningful psychological distress was not significantly elevated during the early or late pandemic periods but was higher in the post-pandemic period when compared with pre-2020 levels. This pattern was most evident for the anxiety subscale and among specific population subgroups, including males, younger adults, and college graduates. Together, these findings reveal important changes in population-level mental health following the COVID-19 pandemic, and highlight a key need for ongoing prevention and intervention efforts, specifically among certain population subgroups.

The higher prevalence of psychological distress observed in 2024 may reflect multiple overlapping social and structural conditions rather than a single pandemic-related mechanism. Potential contributors include lingering social disruption (Topazian et al., 2022), bereavement (Grace, 2021), economic insecurity (Mueller et al., 2022), employment instability (Breslau et al., 2023), caregiving demands (Lee et al., 2024), barriers to physical and mental healthcare access (Zhong et al., 2022), changes in telehealth availability (Molfenter, n.d.), and broader social and political uncertainty (Kwon, 2023). Individual-level factors such as social isolation and problematic social media use may also be relevant (Giovenco et al., 2022). The present analysis was not designed to test these pathways, and thus, these factors should be interpreted as plausible contextual contributors to post-pandemic mental health patterns rather than mechanisms directly supported by the current study.

Although the post-pandemic prevalence of psychological distress was only a modest 2 percentage points higher than pre-pandemic levels, this corresponds to approximately 5.4 million additional US adults experiencing elevated distress on a clinically validated screening instrument, underscoring the need for sustained investment in mental health resources and prevention strategies. Targeted approaches may be particularly important for groups with the largest observed increases, including males and younger adults. These could include interventions to reduce stigma, improve mental health literacy, and expand access to care (SK et al., 2022). The use of telehealth for mental health services increased during the pandemic, but reductions in insurance reimbursement may limit continued access in the post-pandemic period (Molfenter, n.d.). Interventions addressing social isolation and social media use may also be warranted, as both remain prevalent and are associated with anxiety and depression (Zhu et al., 2026; Gottfried, 2024). Previous studies have demonstrated the positive mental health benefits of exercise and introducing scheduled breaks from social media (Tang et al., 2024; Lambert et al., 2022; Carek et al., 2011). Interventions that highlight exercise may be particularly helpful, as they could also serve to reduce other health-related issues associated with increased BMI during the COVID-19 pandemic (Restrepo, 2022).

We acknowledge certain limitations in this study. First, though the PHQ-4 has established reliability and validity as a screening instrument for anxiety and depression, further evaluation by a mental health professional is necessary to obtain a clinical diagnosis. Second, low response rates for HINTS iterations may introduce bias, though all calculations in our study were weighted to account for nonresponse. Third, differences in the mode of survey administration (paper versus online) between different HINTS survey iterations may have impacted findings. However, previous literature comparing results of paper versus online administration of mental health questionnaires have demonstrated strong correlation of findings across modes (Erbe et al., 2016; Muehlhausen et al., 2015). Fourth, educational attainment was used as a proxy for socioeconomic position because household income was missing for over 10% of respondents. Though highly correlated with income levels overall, education does not fully capture economic vulnerability, including income loss, wealth, debt, employment instability, insurance coverage, material hardship, or caregiving burden. Residual confounding by socioeconomic conditions may therefore remain, particularly given that economic insecurity may be closely related to psychological distress during the study period. Fifth, this study is only able to assess changes in mental health among adults. Increases in anxiety and depression among adolescents during the COVID-19 pandemic have been noted elsewhere, though further studies into trends and ongoing impacts within adolescent populations and how they may differ from the adult population are warranted (Hawes et al., 2022; Xiang et al., 2024). Sixth, the post-pandemic period was represented by a single 2024 survey cycle. Although this timing followed the end of the WHO's COVID-19 public health emergency, a single post-pandemic assessment cannot determine whether psychological distress had stabilized, was continuing to increase, or was declining from an unobserved earlier peak. Seventh, causality cannot be established through the use of repeated cross-sectional survey data. Observed differences across periods should not be interpreted as individual-level trajectories or changes directly attributable to the COVID-19 pandemic itself, as other contemporaneous factors—including economic conditions, healthcare access, policy changes, and social or political stressors—may have contributed to psychological distress during the study period. Nevertheless, the greatest strength of this study is its use of nationally-representative survey data spanning multiple periods surrounding the COVID-19 pandemic.

## 5 Conclusions

Analyzing repeated cross-sectional trends in mental health between 2017 and 2024, we found that the prevalence of clinically meaningful psychological distress was higher among US adults after the COVID-19 pandemic than before, with the clearest differences observed for anxiety-related symptoms. The largest group-level changes were observed among males, individuals aged 18–34 years, individuals of non-Hispanic White race and ethnicity, and college graduates. Continued monitoring of population-level psychological distress following the COVID-19 pandemic is important for informing targeted prevention strategies and resource allocation to address ongoing mental health needs.

## CRediT authorship contribution statement

**Todd Burus:** Writing – review & editing, Writing – original draft, Visualization, Validation, Resources, Project administration, Investigation, Formal analysis, Data curation, Conceptualization. **Tia N. Weber:** Writing – review & editing, Writing – original draft, Validation, Supervision, Resources, Project administration, Investigation, Data curation, Conceptualization.

## Declaration of competing interest

The authors declare that they have no known competing financial interests or personal relationships that could have appeared to influence the work reported in this paper.

## Data Availability

Data will be made available on request.
