# Supplementary material for: Trends in psychological distress among US adults before, during, and after the COVID-19 pandemic: a repeated cross-sectional analysis, 2017–2024
Source: Prev Med Rep. 2026 Jun 30;68:103554. doi: 10.1016/j.pmedr.2026.103554 (PMC13334392; doi:10.1016/j.pmedr.2026.103554)
Supplement: Supplementary file 1 — Supplementary material [file mmc1.docx]

**Supplementary Material**

**Supplementary Figure 1.** Study Population Sample Flow Diagram.

**Supplementary Table 1.** Prevalence of clinically-meaningful psychological distress among US adults by period and subgroup, Health Information National Trends Survey, 2017-2024.

**Supplementary Table 2.** Comparison of Patient Health Questionnaire-4 (PHQ-4) results to pre-pandemic period among US adults using hot deck imputation, Health Information National Trends Survey, 2017-2024.

**Supplementary Table 3.** Changes in anxiety disorders by US adult subgroups, Health Information National Trends Survey, 2017-2024.

**Supplementary Table 4.** Changes in depressive disorders by US adult subgroups, Health Information National Trends Survey, 2017-2024.

This supplement has been provided by the authors to give readers additional information about their work.

**Supplementary Figure 1. Study population sample flow diagram.**

**
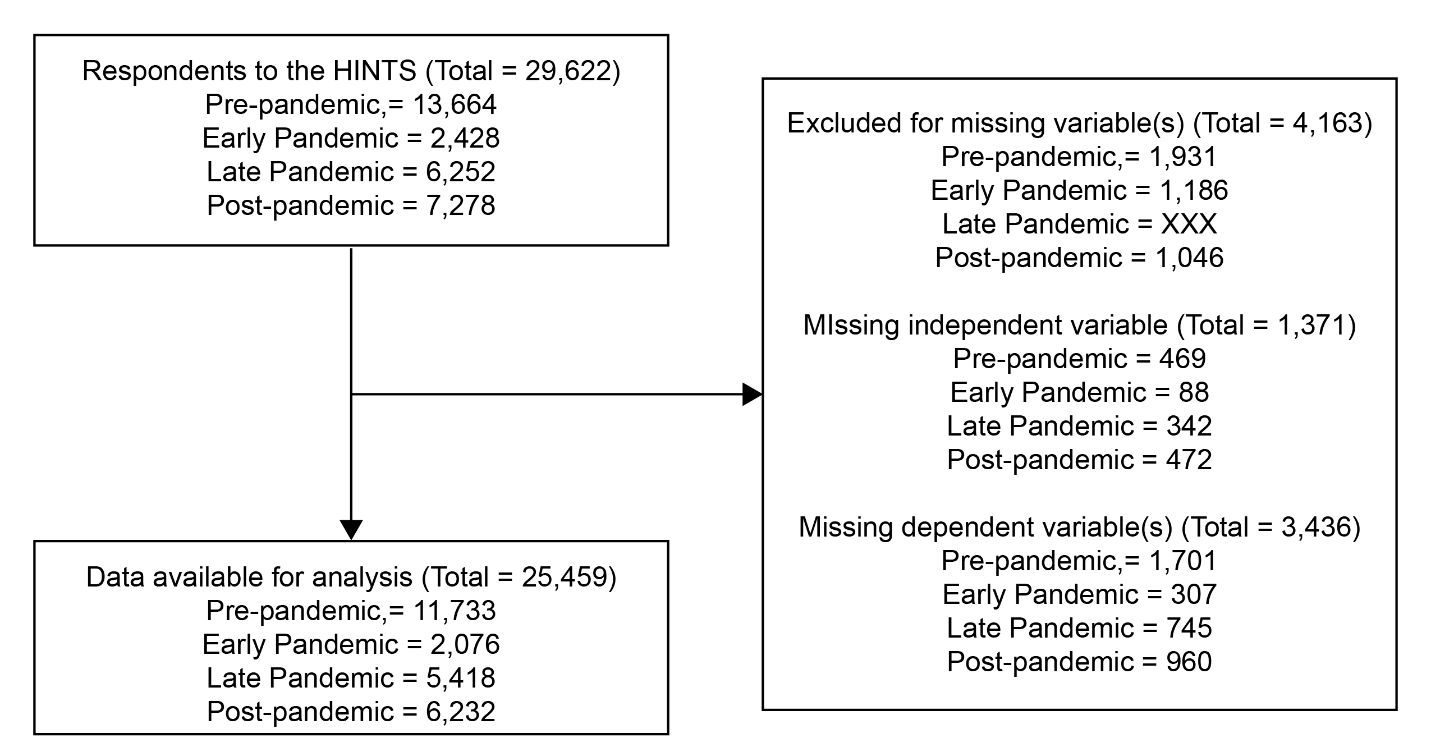
**

**Supplementary Table 1. Prevalence of clinically-meaningful psychological distress among US adults by period and subgroup, Health Information National Trends Survey, 2017-2024**. Pre-pandemic period includes HINTS 5 Cycle 1 (2017), HINTS 5 Cycle 2 (2018), HINTS 5 Cycle 3 (2019), and HINTS 5 Cycle 4 (February 1 - March 11, 2020). Early Pandemic period includes HINTS 5 Cycle 4 (March 12 - June 30, 2020). Late Pandemic period includes HINTS 6 (2022). Post-pandemic period includes HINTS 7 (2024). Abbreviations: CI = confidence interval; Prop = weighted proportion.

| **Group** | **Pre-pandemic,**  **Prop (95%CI)** | **Early Pandemic,**  **Prop (95%CI)** | **Late Pandemic,**  **Prop (95%CI)** | **Post-pandemic,**  **Prop (95%CI)** |
| --- | --- | --- | --- | --- |
| Overall | 12.8% (11.7%,14.0%) | 14.7% (11.4%,18.4%) | 14.0% (12.2%,15.8%) | 15.0% (13.8%,16.2%) |
| Sex |  |  |  |  |
| Female | 15.4% (14%,17%) | 16.3% (12%,21.4%) | 15.2% (13.1%,17.5%) | 12.6% (9.8%,15.9%) |
| Male | 10.2% (8.6%,12%) | 12.9% (8.7%,18.2%) | 12.7% (10.1%,15.6%) | 17.3% (15.5%,19.2%) |
| Age Group |  |  |  |  |
| Aged 18-34 years | 17.2% (13.9%,21%) | 19.1% (11.3%,29.2%) | 22% (17.1%,27.6%) | 24.7% (20.9%,28.7%) |
| Aged 35-49 years | 13.5% (11.1%,16.2%) | 16.4% (11.2%,22.8%) | 16.8% (13%,21.3%) | 15.2% (11.2%,20%) |
| Aged 50-64 years | 12% (10.5%,13.6%) | 11% (7.2%,16%) | 9.8% (7.8%,12%) | 11.7% (9.3%,14.4%) |
| Aged 65-74 years | 7.2% (5.9%,8.7%) | 7.3% (4%,12%) | 6.3% (4.4%,8.6%) | 5.9% (4%,8.3%) |
| Aged ≥75 years | 8.1% (6.1%,10.5%) | 10.9% (4.8%,20.3%) | 6.4% (4.1%,9.4%) | 7.9% (4.8%,11.9%) |
| Race and Ethnicity |  |  |  |  |
| Hispanic | 15.5% (12.6%,18.8%) | 14.9% (9.2%,22.3%) | 17.8% (12.7%,23.9%) | 19.5% (16.3%,22.9%) |
| Non-Hispanic Asian | 10.9% (5%,19.9%) | 5.3% (1.3%,13.8%) | 8.8% (4.1%,16%) | 6.8% (3.2%,12.4%) |
| Non-Hispanic Black | 13.3% (10.4%,16.7%) | 12.1% (6.5%,19.8%) | 15.4% (10.9%,20.9%) | 13.7% (10.4%,17.5%) |
| Non-Hispanic White | 12.1% (10.7%,13.5%) | 15.5% (11.7%,19.9%) | 12.9% (10.7%,15.4%) | 14.2% (12%,16.6%) |
| Other Non-Hispanic Races | 17.4% (10%,27.4%) | 27.6% (8.6%,55.3%) | 16.8% (8.4%,28.6%) | 19.5% (10.4%,31.8%) |
| Education |  |  |  |  |
| No College | 15.7% (13.7%,18%) | 17.6% (11.1%,25.9%) | 16.2% (12.5%,20.6%) | 21.3% (17.5%,25.5%) |
| Some College | 14.6% (12.6%,16.8%) | 13.4% (9.1%,18.7%) | 14.4% (11.3%,17.9%) | 13.9% (11.8%,16.1%) |
| College Graduate | 8.3% (6.9%,9.9%) | 13.4% (9.4%,18.3%) | 11.6% (9.8%,13.6%) | 11% (9.3%,13%) |
| Urbanicity |  |  |  |  |
| Metropolitan | 12.7% (11.4%,14%) | 15% (11.6%,19%) | 13.4% (11.7%,15.2%) | 14.6% (12.9%,16.3%) |
| Nonmetropolitan | 14.1% (11.3%,17.2%) | 11.9% (6.6%,19.3%) | 18.2% (13.8%,23.3%) | 17% (12.4%,22.5%) |
| Census Region |  |  |  |  |
| Northeast | 11% (8.6%,13.7%) | 12.2% (6.5%,20.3%) | 13.4% (9.6%,18.1%) | 12.2% (9.2%,15.8%) |
| Midwest | 11.8% (9.4%,14.6%) | 15.2% (8.6%,24.2%) | 12.1% (9.3%,15.4%) | 13.7% (10.3%,17.7%) |
| South | 14% (12.1%,16.1%) | 15.9% (11.2%,21.5%) | 15.5% (12.7%,18.6%) | 17.4% (14.5%,20.6%) |
| West | 13.3% (11%,16%) | 14% (9%,20.4%) | 13.6% (10.1%,17.6%) | 13.9% (11.1%,17%) |

**Supplementary Table 2.** **Comparison of Patient Health Questionnaire-4 (PHQ-4) results to pre-pandemic period among US adults using hot deck imputation, Health Information National Trends Survey, 2017-2024.** Adjusted prevalence ratio (aPR) of PHQ-4 results during the COVID-19 pandemic and post-pandemic periods compared to the pre-pandemic period, adjusted for sex, age, race and ethnicity, educational attainment, urbanicity, and Census region residence. Missing covariate data imputed using hot deck imputation with 20 replications. Pre-pandemic period includes HINTS 5 Cycle 1 (2017), HINTS 5 Cycle 2 (2018), HINTS 5 Cycle 3 (2019), and HINTS 5 Cycle 4 (February 1 - March 11, 2020). Early Pandemic period includes HINTS 5 Cycle 4 (March 12 - June 30, 2020). Late Pandemic period includes HINTS 6 (2022). Post-pandemic period includes HINTS 7 (2024). Abbreviations: CI = confidence interval.

|  | **Early Pandemic  (aPR, 95% CI)** | **Late Pandemic  (aPR, 95% CI)** | **Post-pandemic (aPR, 95% CI)** |
| --- | --- | --- | --- |
| **Status** |  |  |  |
| Clinically meaningful psychological distress (PHQ-4 ≥ 6) | 1.06 (0.85,1.32) | 1.04 (0.90,1.21) | 1.14 (1.01,1.30) |
| Positive anxiety disorder  (anxiety subscale ≥ 3) | 1.07 (0.90,1.27) | 1.12 (0.98,1.26) | 1.17 (1.04,1.32) |
| Positive depressive disorder  (depression subscale ≥ 3) | 1.02 (0.83,1.24) | 1.10 (0.97,1.25) | 1.12 (0.99,1.27) |

**Supplementary Table 3. Changes in anxiety disorders by US adult subgroups, Health Information National Trends Survey, 2017-2024.** Adjusted prevalence ratio (aPR) of individuals with Patient Health Questionnaire-4 anxiety subscale score ≥ 3 during the COVID-19 pandemic and post-pandemic periods compared to the pre-pandemic period, adjusted for sex, age, race and ethnicity, educational attainment, urbanicity, and Census region residence. Pre-pandemic period includes HINTS 5 Cycle 1 (2017), HINTS 5 Cycle 2 (2018), HINTS 5 Cycle 3 (2019), and HINTS 5 Cycle 4 (February 1 - March 11, 2020). Early Pandemic period includes HINTS 5 Cycle 4 (March 12 - June 30, 2020). Late Pandemic period includes HINTS 6 (2022). Post-pandemic period includes HINTS 7 (2024). Abbreviations: CI = confidence interval.

| **Group** | **Early Pandemic**  **(aPR, 95% CI)** | **Late Pandemic**  **(aPR, 95% CI)** | **Post-pandemic**  **(aPR, 95% CI)** |
| --- | --- | --- | --- |
| Sex |  |  |  |
| Female | 1.00 (0.79,1.25) | 1.11 (0.95,1.28) | 0.85 (0.68,1.05) |
| Male | 1.21 (0.87,1.70) | 1.27 (1.02,1.58) | 1.77 (1.48,2.12) |
| Age Group |  |  |  |
| Aged 18-34 years | 1.00 (0.70,1.44) | 1.17 (0.90,1.51) | 1.39 (1.10,1.75) |
| Aged 35-49 years | 1.39 (1.01,1.93) | 1.38 (1.08,1.77) | 1.17 (0.90,1.52) |
| Aged 50-64 years | 0.90 (0.64,1.26) | 1.07 (0.87,1.31) | 1.15 (0.93,1.43) |
| Aged 65-74 years | 0.91 (0.56,1.48) | 1.11 (0.84,1.48) | 0.96 (0.62,1.48) |
| Aged ≥75 years | 1.33 (0.72,2.46) | 0.78 (0.50,1.21) | 1.11 (0.68,1.80) |
| Race and Ethnicity |  |  |  |
| Hispanic | 1.09 (0.74,1.61) | 1.21 (0.90,1.64) | 1.32 (1.04,1.68) |
| Non-Hispanic Asian | 1.04 (0.33,3.24) | 1.11 (0.44,2.78) | 1.30 (0.56,3.02) |
| Non-Hispanic Black | 0.84 (0.51,1.40) | 1.19 (0.86,1.63) | 1.08 (0.80,1.45) |
| Non-Hispanic White | 1.14 (0.89,1.48) | 1.15 (0.97,1.38) | 1.23 (1.03,1.46) |
| Other Non-Hispanic Races | 1.31 (0.64,2.70) | 1.22 (0.65,2.27) | 0.91 (0.49,1.70) |
| Education |  |  |  |
| No College | 0.99 (0.69,1.44) | 1.05 (0.83,1.33) | 1.37 (1.12,1.69) |
| Some College | 1.05 (0.74,1.48) | 1.12 (0.90,1.40) | 1.01 (0.83,1.24) |
| College Graduate | 1.35 (1.02,1.79) | 1.47 (1.22,1.76) | 1.42 (1.15,1.75) |
| Urbanicity |  |  |  |
| Metropolitan | 1.13 (0.91,1.39) | 1.17 (1.02,1.34) | 1.20 (1.05,1.37) |
| Nonmetropolitan | 0.75 (0.45,1.25) | 1.21 (0.92,1.60) | 1.27 (0.89,1.82) |
| Census Region |  |  |  |
| Northeast | 1.06 (0.64,1.74) | 1.37 (0.96,1.98) | 1.27 (0.90,1.78) |
| Midwest | 1.29 (0.85,1.94) | 1.16 (0.88,1.54) | 1.28 (0.95,1.73) |
| South | 1.13 (0.82,1.55) | 1.20 (0.99,1.45) | 1.21 (1.00,1.47) |
| West | 0.88 (0.57,1.35) | 1.03 (0.78,1.36) | 1.07 (0.86,1.33) |

**Supplementary Table 4. Changes in depressive disorders by US adult subgroups, Health Information National Trends Survey, 2017-2024.** Adjusted prevalence ratio (aPR) of individuals with Patient Health Questionnaire-4 depression subscale score ≥ 3 during the COVID-19 pandemic and post-pandemic periods compared to the pre-pandemic period, adjusted for sex, age, race and ethnicity, educational attainment, urbanicity, and Census region residence. Pre-pandemic period includes HINTS 5 Cycle 1 (2017), HINTS 5 Cycle 2 (2018), HINTS 5 Cycle 3 (2019), and HINTS 5 Cycle 4 (February 1 - March 11, 2020). Early Pandemic period includes HINTS 5 Cycle 4 (March 12 - June 30, 2020). Late Pandemic period includes HINTS 6 (2022). Post-pandemic period includes HINTS 7 (2024). Abbreviations: CI = confidence interval.

| **Group** | **Early Pandemic**  **(aPR, 95% CI)** | **Late Pandemic**  **(aPR, 95% CI)** | **Post-pandemic**  **(aPR, 95% CI)** |
| --- | --- | --- | --- |
| Sex |  |  |  |
| Female | 0.96 (0.74,1.24) | 1.09 (0.93,1.27) | 0.96 (0.77,1.19) |
| Male | 1.13 (0.77,1.65) | 1.20 (0.94,1.52) | 1.35 (1.08,1.67) |
| Age Group |  |  |  |
| Aged 18-34 years | 1.13 (0.67,1.89) | 1.53 (1.14,2.05) | 1.39 (1.05,1.85) |
| Aged 35-49 years | 1.08 (0.73,1.59) | 1.10 (0.83,1.45) | 1.07 (0.78,1.47) |
| Aged 50-64 years | 0.82 (0.57,1.18) | 0.80 (0.63,1.01) | 1.07 (0.85,1.35) |
| Aged 65-74 years | 1.01 (0.63,1.60) | 1.14 (0.85,1.53) | 0.92 (0.66,1.27) |
| Aged ≥75 years | 1.62 (0.94,2.80) | 1.25 (0.93,1.69) | 0.95 (0.62,1.45) |
| Race and Ethnicity |  |  |  |
| Hispanic | 0.73 (0.48,1.10) | 1.23 (0.90,1.69) | 1.25 (0.97,1.62) |
| Non-Hispanic Asian | 0.63 (0.25,1.54) | 0.97 (0.49,1.91) | 0.49 (0.22,1.11) |
| Non-Hispanic Black | 0.77 (0.46,1.27) | 1.11 (0.80,1.55) | 1.01 (0.74,1.37) |
| Non-Hispanic White | 1.27 (0.96,1.67) | 1.16 (0.97,1.39) | 1.14 (0.93,1.40) |
| Other Non-Hispanic Races | 1.63 (0.56,4.77) | 0.84 (0.36,1.95) | 1.37 (0.73,2.56) |
| Education |  |  |  |
| No College | 1.04 (0.73,1.49) | 1.07 (0.86,1.34) | 1.16 (0.93,1.46) |
| Some College | 0.86 (0.61,1.21) | 1.05 (0.83,1.32) | 1.01 (0.81,1.27) |
| College Graduate | 1.44 (1.01,2.05) | 1.47 (1.21,1.79) | 1.33 (1.06,1.67) |
| Urbanicity |  |  |  |
| Metropolitan | 1.03 (0.81,1.32) | 1.12 (0.97,1.31) | 1.10 (0.95,1.29) |
| Nonmetropolitan | 0.96 (0.53,1.74) | 1.21 (0.88,1.66) | 1.23 (0.86,1.76) |
| Census Region |  |  |  |
| Northeast | 1.20 (0.69,2.09) | 1.42 (1.02,1.99) | 0.99 (0.70,1.40) |
| Midwest | 1.04 (0.63,1.71) | 1.06 (0.79,1.43) | 1.15 (0.82,1.60) |
| South | 1.00 (0.71,1.41) | 0.99 (0.80,1.22) | 1.15 (0.94,1.41) |
| West | 0.96 (0.61,1.49) | 1.29 (0.99,1.69) | 1.10 (0.84,1.43) |
